# Silent wounds: violence and adverse experiences in parents caring for terminally ill children in palliative care

**DOI:** 10.1186/s12904-026-02134-9

**Published:** 2026-05-11

**Authors:** Jessica Guadarrama-Orozco, Martha Romero-Mendoza, Maria Gabriela Mendoza-Martínez, Ingris Peláez-Ballestas, Amaranta Manrique de Lara, Michelle Muñoz-Pedraza

**Affiliations:** 1 Department of Palliative Care and Quality of Life, Hospital Infantil de México “Federico Gómez”, Mexico City, México; 2 Direction of Psychosocial and Epidemiological Research, Instituto Nacional de Psiquiatría “Ramón de La Fuente Muñiz”, Mexico City, Mexico; 3 Rheumatology Unit, Hospital General de México “Dr. Eduardo Liceaga”, Mexico City, Mexico; 4 Research Unit, Hospital General de México “Dr. Eduardo Liceaga”, Mexico City, Mexico

**Keywords:** Palliative medicine & chronic care, Quality of life, Paediatrics, Caregivers, Violence, ACES

## Abstract

**Background:**

Primary caregivers of paediatric palliative care patients—mostly women—are disproportionately exposed to adverse events and violence, both directly related to the experience of disease and to other stressful life events. These experiences have been associated with a higher risk of psychopathology, which in turn impacts the children under their care. The purpose of this study was to describe the experiences of violence among primary caregivers of palliative care patients in a children’s hospital in Mexico City and to analyse how these experiences affect their caregiving capacity.

**Methods:**

A qualitative study was conducted in which narratives were reconstructed from semi-structured interviews with primary caregivers, non-participant observations, and hospital documentation. Caregivers from 21 families with a child receiving palliative care participated in the study. Data analysis was performed using Atlas.ti software, with further triangulation between researchers and a multidisciplinary team of health professionals.

**Results:**

Most caregivers narrated multiple and diverse experiences of violence across the lifespan, interwoven with intergenerational impacts. Experiences of violence were categorized into four groups: adverse childhood experiences, stressful life events in adulthood, onset of disease in the child, and consequences of disease. Violence was frequent among participants, and exposure to constant stressful life events had a negative impact on their ability to cope with adverse experiences, including the child’s illness.

**Conclusions:**

Recognizing and addressing the violence experienced by female caregivers is essential to paediatric palliative care practice. Prevention of re-traumatization and vicarious trauma among patients, families, and providers is needed to improve therapeutic interactions. Individualized interventions for survivors of violence could reduce the burden of disease on caregivers and disrupt intergenerational cycles of violence.

**Supplementary Information:**

The online version contains supplementary material available at 10.1186/s12904-026-02134-9.

## Introduction

Caring for children and adolescents with severe health problems can be stressful work, commonly leading to tensions like disruptions in employment, financial strain, difficulties in transportation, and changes to the family routine and dynamic, the family plays a crucial role in enhancing the quality of life for these children. The emotional stress and burdens experienced by the caregiver are associated with diminished quality of life and conflict within the family [1]. Existing research suggests that financial strain along with the sociodemographic characteristics of the caregiver like race/ethnicity, marital status, schooling—can be predictors of the pressure and emotional burden faced in care work [2].

Palliative care encompasses the health services that aim to ease the suffering of patients with progressive, incurable diseases, through the fulfilment of their physical, psychological, social and existential needs, including support services for caregivers [3]. The caregiver’s role in palliative care is both emotionally and physically challenging because they frequently perform two different roles at the same time: as care providers to the patient and as a link between the patient and health services [3]. However, what is often forgotten is that the caregiver is also a person with their own feelings and needs, in the case of children, the caregivers are usually their parents.

Caregivers in palliative care services—particularly paediatric palliative care (PPC)—tend to be women, who are at twice the risk of emotional distress and care overload throughout their lives than men. Additionally, since women spend over 8 h everyday performing care work, they often must leave their employment, and the loss of income leads to increased financial strain. Some factors further complicate care work: The time and intensity of care increases as a patient’s mobility decreases, or if the patient presents symptoms such as dyspnea, depression and anxiety. Furthermore, a caregiver is forced to change their entire lifestyle when the child is close to the end-of-life, and care burdens are increased in accordance with the number of visits to urgent care services [4]. Palliative care aims to improve the quality of life of both patients and their caregivers by alleviating the social, spiritual, and even economic burdens associated with serious illness [5]. However, this goal cannot be fully achieved without acknowledging that each caregiver’s may have their own adverse life experiences—including exposure to violence, childhood adversity, and ongoing psychosocial stressors—inevitably shape the way in which they provide care to the seriously ill child. A caregiver who carries the weight of unaddressed trauma may struggle to meet not only their own needs but also the complex demands of caring for a child with a life-limiting condition, ultimately affecting the quality and consistency of that care.

Though the symptoms experienced by children—such as pain, sleep disorders, and emotional and behavioral issues—impact caregiver strain, there is a lack of research about their influence or about caregiver experiences in general throughout the course of the disease and suffering of a child. Similarly, there is just a small number of studies about caregivers’ adverse experiences during childhood, intimate partner violence, gender-based violence, and the neglect of siblings in favor of caring for the ill child. Moreover, little research has explored how caregivers feel about these experiences and whether they can impact the quality of care provided [6, 7].

Adverse childhood experiences (ACEs) are preventable, potentially stressful and traumatic events that occur during childhood and adolescence. They encompass diverse aspects of family dysfunction, such as sexual, physical and emotional abuse; physical neglect; bearing witness to violence, suicide or imprisonment of a family member; etc. These experiences cause suffering and can even lead to post-traumatic stress, diminishing a child’s sense of safety, stability and attachment, consequently impacting development and growth [8–11].

ACEs have been associated with a higher risk of pathology throughout the lifetime and of chronic stress related to health problems; therefore, ACEs impact life chances [12]. This is further aggravated in the context of social adversity, such as food, housing and job insecurity, poverty, lack of social support, and economic and racial segregation [13]. Currently, evidence suggests that maternal and paternal ACEs can affect the health and development of children, and this intergenerational impact is associated with epigenetic changes. Studies link maternal ACEs with psychopathologies in children, decreased behavioral development, weakened relationship between parents and children due to an environment of control and fear, problems in upbringing, isolation of the family unity, higher probability of abuse or neglect, and social adversity [11, 14, 15].

Additionally, ACEs have been associated with a higher probability of miscarriages, unwanted pregnancies, decreased prenatal mental health, intimate partner violence during pregnancy [1], and substance abuse during pregnancy, among other problems [9].

When there is a lack of protective factors or these are uncertain, the conditions of emotional and material deprivation lead to the accumulation of toxic stress which negatively affects the neuroendocrinal system and brain development [16]. Research in neuroscience has generated overwhelming evidence regarding the biological mechanisms that can alter the architecture of neural development in the child, leading to diminished capabilities for learning and memory retention, lack of self-regulation, and excessive functionality [10].

Violence against women has been defined as any act of violence that results in physical, sexual or mental harm towards women, including threats of these acts of violence, occurring in private or in public life [17]. Similarly, intimate partner violence refers to behaviors by a partner or former partner that cause physical, sexual or psychological distress, including physical violence, sexual coercion, psychological abuse, and controlling behaviors, among others [17].

**Fig. 1:**
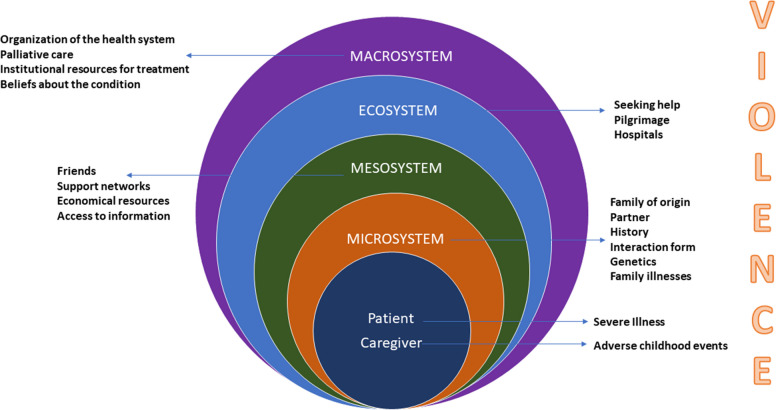
Ecological model of pediatric palliative care and violence

Both ACEs and experiences of gender-based violence can be traumatic, organized within social, familial and cultural realities that permanently and reciprocally interact with each other (Fig. 1) [18]. This trauma is a harmful and costly public health issue, resulting from violence, abuse, negligence, loss, disasters, wars, and other negative emotional experiences [19].

From a sociopolitical standpoint, ACEs and gender-based violence entail an exercise of power. This is defined as the ability, energy or strength to do, cause or prevent something that’s considered beneficial or not; it’s the ability to impose one’s own will over others so they do or abstain from something, or so they directly or indirectly accept something that they would otherwise reject [20]. Power is exercised by taking advantage of helplessness, inability, indifference, complicity or fear of others.

The objective of this study was thus to describe the ACEs and experiences of intimate partner and gender violence, as well as analyze how these impact coping and care provision, of primary caregivers of PPC patients in a children’s hospital in Mexico City.

## Methods

This was a case study, defined as a qualitative approach in which the research explores a topic within a real-life situation. In-depth data is collected from diverse sources (ethnographic observation, semi-structured interviews, documents, and reports), resulting in a description of the case and the relevant associated themes. This research is conducted in a single site and entails an intrinsic case, as it presents a unique situation [21].

The intrinsic case study design “is an intrinsic case study in which the focus is on the case itself because the case presents an unusual or unique situation. This resembles the focus of narrative research, but the case study analytic procedures of a detailed description of the case, set within its context or surroundings, still hold true.” ([21], p.99) Therefore, the aim of adopting this approach was not to construct a theory or generalize results, but to generate a potential benefit for individuals based on the singular experiences and contexts, in this case families traversing through pediatric palliative care, improving their relationship with the child or providing support during grief.

### Study design

A thematic analysis [22] was conducted in six stages: 1) familiarization with the narratives, 2) coding, 3) theme generation, 4) theme development and revision, 5) refinement and definition, and 6) naming the themes and writing. In this last stage, relevant quotes and fragments from the data are interwoven with an analytical narrative, written in a way that is aligned with the research’s paradigm and values, to achieve the study’s objective.

### Interview guide

We constructed an interview guide to explore aspects related to the objectives of the study. The guide was based on a non-systematic literature review, as well as the researchers’ own experiences in the PPC service. Some of the themes explored were conceptualization of disease and care; causes, symptoms and consequences of disease; chronological sequence in the search for care; types of care and personal experiences with care; experience at the hospital; religion; conceptualization of palliative care; emotional and financial impact; and perceptions of time, the future and quality of life (Supplementary files 1).

### Research strategy

We conducted a pilot study before the start of the interviews to test the guide, standardize the interviewers and test the study strategy. The pilot interview was not included in the final analysis.

Primary caregivers of children seen by the PPC service of a public specialized children’s hospital were invited to participate. This hospital is a public institution reaching uninsured children around the country who require highly specialized care, particularly those from Mexico’s central region.

The medical staff of the palliative care department invited families to participate, by phone or in person, depending on each family’s specific situation. Afterwards, an appointment was set at the hospital to present the project, address questions, and allow invitees to decide whether to participate. Interviews were scheduled according to each participant's availability.

We conducted interviews in the hospital, in a comfortable and private office. The interviews, including informed consent, were recorded in duplicate using a voice recorder and a mobile phone. Three researchers completed the interviews. Afterwards, one of the interviewers and a social worker from the PPC service transcribed the interviews using a word processor.

The interviews were complemented with information obtained during hospital rounds, observation of the palliative care service and clinical and sociodemographic records (reviewed by MGMM, JGO). All of these were systematically compiled into field notes and included in the interview contexts for the analysis.

### Data collection

Participants were recruited through convenience sampling, based on the following inclusion criteria: (1) being the primary caregiver of a child actively followed by the PPC service, (2) having been under PPC follow-up for at least one month, and (3) willingness to participate in an interview. No exclusion criteria were applied. All children were alive at the time of the study, either at home or hospitalized. All invited caregivers chose to participate in the study.

### Ethical considerations

Once participants accepted, the informed consent form was read and explained in detail, including the study title, justification, objectives, procedures, expected risks and discomfort and potential benefits. Participants were able to ask questions about the research process and receive clarification from the PPC service. Participants were informed that they were able to withdraw consent at any point during the interview and study, and that confidentiality and anonymity were ensured since only the research team would have access to the interviews.

Given the participants’ condition of vulnerability and the sensitive nature of the themes covered, support strategies were implemented during and after the interviews. All three interviewers hold formal training in psychological first aid and emergency emotional containment, enabling them to recognize and respond to signs of distress in real time. Additionally, a response protocol was in place so that qualified personnel and/or support networks linked with the PPC service (psychologists, thanatologists) could immediately intervene if necessary. This emphasis on emotional and psychosocial support is a core and continuous pillar of the PPC service, so that caregivers had access to previous and ongoing follow-up by a psychologist specialized in pediatric palliative care throughout the entire course of their child’s illness, regardless of their participation in the study. This included access to grief workshops and structured bereavement care.

### Sampling

Participants were 21 primary caregivers from 21 families with a child in the PPC service. 20 (95%) were women, with a mean age of 35 and a mode of completed secondary school for both parents. The average income per family was USD$ 344 a month, with a maximum income of USD$ 1060 and a minimum of USD $85 a month. The children’s diagnoses were diverse. Other important data can be found in Tables 1 and 2.

**Table 1:** Characteristics of primary caregivers

Patient code	Age	Gender	Schooling	Occupation	Relation to patient
J.A_1	30	F	Primary school (unfinished)	Housewife	Mother
J.L_2	29	F	Secondary school	Housewife	Mother
L_3	29	F	University	Catalog sales representative	Mother
Z_4	34	F	High school	Housewife	Mother
L_5	39	F	Secondary school	Housewife	Mother
D.G_6	41	F	Primary	Housewife	Mother
C_7	25	F	Secondary school	Farmer	Mother
V_8	33	F	Primary	Saleswoman (desserts)	Mother
D_9	37	F	University	Housewife	Mother
O_10	43	M	Primary	Taxi driver	Father
L.C_11	30	F	Secondary school	Housewife	Mother
S.Y_12	54	F	Secondary school	Saleswoman (apparel)	Maternal grandmother
J.Q_13	29	F	High school	Housewife	Mother
L_14	29	F	University	Teacher	Mother
A.P_15	35	F	Secondary school	Housewife	Mother
A.G_16	52	F	Primary	Saleswoman	Paternal grandmother
E_17	29	F	Secondary school	Saleswoman	Mother
N_18	21	F	Secondary school	Teacher	Mother
C_19	31	F	Secondary school	Housewife	Mother
J_20	41	F	Secondary school	Artisan trader	Mother
A.D_21		F	University	Employee	Maternal aunt

**Table 2:** Patients clinical data

Patient code	Age/months	Gender	Diagnosis	PC date service entry	Time in PC	Dead or alive	Date of death
J.A_1	107	M	Pulmonary fibrosis	28.10.21	3y 8 m	Alive	
J.L_2	100	M	Neuroblastoma (adrenal primary) with vitreous hemorrhage	16.10.19	2y 4 m	Death	02.02.22
L_3	24	F	Medulloblastoma, Focal Epilepsy, Ventriculitis	18.11.21	10 m	Death	21.09.22
Z_4	151	F	Osteoblastic Osteosarcoma left distal femur	06.08.21	7 m	Death	16.03.22
L_5	39	M	Pulmonary artery sling	28.12.20	4y 6 m	Alive	
D.G_6	191	M	Acute myeloid leukemia, pulmonary aspergillosis	30.07.21	5 m	Death	02.12.21
C_7	79	F	Acute lymphoblastic leukemia, mucormycosis	05.05.21	10 m	Death	20.03.22
V_8	16	F	Dandy Walker Syndrome, trisomy 9, hydrocephalus	20.09.21	6 m	Death	10.02.22
D_9	69	M	Duchenne muscular dystrophy	07.12.20	4y 6 m	Alive	
O_10	62	M	Prostate Rhabdomyosarcoma	04.10.21	4 m	Death	25.02.22
L.C_11	63	F	Focal structural epilepsy, bilateral leukomalacia	10.11.21	3y 7 m	Alive	
S.Y_12	108	F	Guillain-Barré syndrome, spastic cerebral palsy	19.02.21	4y 4 m	Alive	
J.Q_13	28	M	Noonan syndrome	08.06.21	4y	Alive	
L_14	5	M	Spinal muscular atrophy type 1	02.12.21	2y 9 m	Death	08.09.24
A.P_15	125	F	Partial symptomatic epilepsy, cerebral palsy	09.10.19	4y 1 m	Death	04.11.23
A.G_16	101	M	Neurological regression syndrome	13.12.21	3y 2 m	Death	03.01.25
E_17	81	F	SLE, lupus nephritis, high blood pressure	20.12.21	3y 6 m	Alive	
N_18	65	M	Wolf-Hirschhorn syndrome, cleft lip/palate, epilepsy	11.10.20	4y 4 m	Death	17.02.24
C_19	13	F	Premature birth 26 wks, neonatal sepsis, IVH G4	29.12.20	4y 6 m	Alive	Counter-ref
J_20	89	M	High risk mediastinal Neuroblastoma	19.05.21	4y 3 m	Death	18.08.24
A.D_21	202	M	Fetal distress, hydrocephalus, cerebral palsy, epilepsy	19.01.22	Discharge 13.03.25	Alive	Medical discharge

Interviews lasted an average of 68 min, with a minimum of 30 min and a maximum of 1 h and 53 min. We only interviewed each participant once.

### Data analysis

All interviews (100%) were fully analyzed and independently coded by three of the researchers (MRM, MGMM, IPB), involving a constant rereading of the interviews and comparison between perspectives.

Following careful reading of the transcripts, a series of codes were created and assigned to different categories with specific characteristics. These were subsequently analyzed at a higher level of abstraction, considering their potential relationships.

Meetings were held between the researchers throughout the analysis, which served to identify and discuss new emerging themes that had not been included in the interview guide but that were present in the participants’ narratives.

Of these, the topic of violence was particularly relevant, given the overwhelming discussion by participants. All the information related to violence was extracted from the rest through a narrative and thematic analysis using Atlas.ti software [23] as a tool for organizing and administrating material in a creative and systematic way. Thematic saturation was reached when no new codes or themes were identified and their contents were redundant [24].

The researchers met with the head of the PPC service to discuss the emergence of this theme and its implications. Afterwards, another researcher was invited to give a presentation about the emerging themes in a two-hour in-person meeting with the PPC service. Twenty health professionals (doctors, nurses, social workers, and bioethicists) participated and agreed that the findings about violence resonated with their experiences and daily interactions with caregivers; they concurred that addressing this topic would be important and impactful.

Any discrepancies in codification and interpretation between the researchers were resolved in discussion groups.

## Results

Most of the participating caregivers narrated difficult family experiences, related to the socioeconomic context they grew up in, as well as to the dynamics with their intimate partners or their families at the time of the child’s disease onset.

**Fig. 2:**
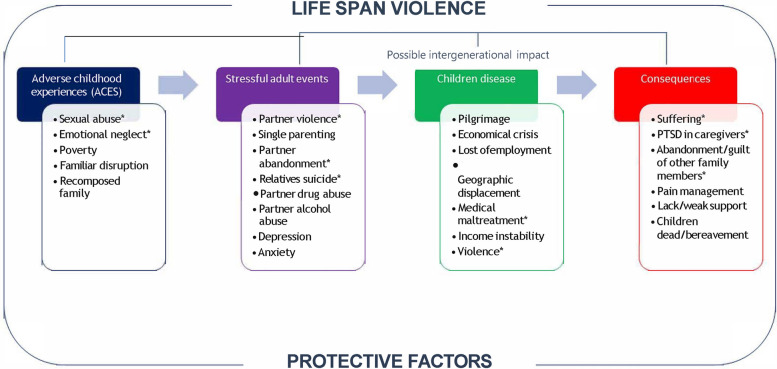
Diachronic and synchronic experiences throughout the course of the disease

The themes identified during the analysis were interpreted based on the concept of Lifespan Violence (Fig. 2), which describes the different stages of a person’s life (childhood, adulthood, and the process of caring for a child in PPC) where violence can manifest. Importantly, this concept initially arises in the context of violence against women as an inherent and complex issue, in which violence occurs across the lifespan and in which these experiences of violence are linked. There is significant evidence to suggest that many adult women who experience violence report multiple forms of victimization, with experience of family violence now recognized as a risk factor for experiencing abuse in different populations (children and elderly people) [25, 26]. We have chosen this interpretative lens due to the overwhelming feminization of care, but also to extend its use beyond violence against women and include other vulnerable populations such as caregivers.

We use this diagram (Fig. 2), to describe diachronic (experienced over time by the caregiver) and synchronic (experienced simultaneously with the child) developments throughout the course of the disease. That is, violence occurs throughout a caregiver’s lifespan concurrently with four groups of experiences that make it more complex and present new challenges for coping: 1) Adverse childhood experiences; 2) Stressful life events in adulthood; 3) Onset of disease in the child; and 4) Consequences of disease. Each group encompasses recurring themes mentioned by caregivers.

### Adverse childhood events

Unfavorable experiences during childhood can be diverse, encompassing emotional, physical or sexual abuse, negligence, substance use, domestic issues, criminal activity, financial adversity, absence of a parent, and abandonment. Additionally, loss, frequent change of residence, and natural disasters can also have an impact.

Z-4: "Yes, but when I was born my parents separated, they each made their own home. I was abandoned."

Many of the women we interviewed narrated experiences and/or suspicions of sexual abuse.

*J.L. 2: "When I was basically like 9 or 11 years old, when I was in primary school, my stepfather was very violent towards my mom and especially towards me. He abused me and he was always threatening to burn my school supplies. He took me to work out in the fields and, well, that’s where everything that happened happened. It was a lot of violence towards my mom; my stepfather beat her a lot. I was very scared, I grabbed a rock and hit him on the head, but even then, he wouldn’t let me go, he clenched my hand. I told my mom, and she didn’t believe me…she said nothing was true. One day my grandparents went to visit my mom, I told my grandparents, and I left. From 14 to 20 I didn’t visit my mom at her house, never ever. It truly was a nightmare; it was very hard for me because I couldn’t even sleep…so many years. I’m still grieving today. Why me? Why did I have to live like that, and I still don’t understand?".*

### Stressful life events in adulthood

In addition to ACEs, caregivers can face events that lead to emotional instability in their adulthood, death and loss in particular.

It’s common for children with severe health problems to be left vulnerable after the loss of a biological parent because of violent death or suicide, or if a parent faces legal issues or imprisonment due to a violent crime. When this happens, legal guardianship is transferred to other family members, mostly grandparents or aunts/uncles, who themselves must navigate vulnerability due to the unexpected loss itself as well as the suddenly acquired responsibility for a child.

*A.E. 16: "He fell into a severe depression, he took his own life, committed suicide, he hung himself in his house when he lived with her…I’ve gone through difficult times…He left me two small persons that are part of him…My son was everything to me, I tried to take my own life after the loss of my son because he was everything to me. And it was very difficult to accept. I’ve accepted it now…there are three children that need me. My son died when… [my grandson] was two months old, when the woman left the kids."*

*A.P._15: "Well, it’s somewhat difficult because in fact, we just had a very intense event. My daughter wanted to commit suicide, and then she came here. We also spent time wandering around the hospital because when I got home from there, I started doing laundry. When I went upstairs to check on her, I came back down, and my daughter said to me, ‘Mom, I feel bad.’—‘What’s wrong?’—‘My face is going numb.’ ‘But what did you do, what did you take?’—‘I took all my pills.’—‘Why did you do that?’—‘It’s just that you only pay attention to my sister, and I also exist.’—‘Yes, daughter, but you shouldn’t have done that.’ And she started to get worse."*

Then, talking about the children’s mother:

*A.E. 16: "Their mother who is in prison told me that it was because they kidnapped a person in their home, they broke in to steal, she and three other people. She got 15 years in prison, so to me that woman is nothing and she’s less to my children, because they’re my children, I take care of them, I live for them, I’m there for the good and bad times, and as long as I have strength and life I’m going to be there for them, I’m going to help them get ahead…I love them, you don’t know how much I truly love those children."*

#### Violent death

The family’s context can be characterized by an unsafe and violent environment.

*A_P15 [about her partner]: "No, I mean he died during a mugging. It was 16 years ago that he was mugged, he lost his life. She was about to turn 1, a month after he was murdered. She was so little."*

#### Intimate partner violence

Intimate partner violence encompasses a set of abusive behaviors such as battering, screaming, non-consensual sexual behaviors, lack of financial support, threats, confinement, and isolation, among many others. In addition to poverty and lack of social solidarity, these behaviors leave the caregiver defenseless against their abuser, commonly resulting in silence and shame.

*J.L.2: "When I was 20 years old, I got together with my son’s father. But one month into the relationship the was a lot of violence, a lot of beating, a lot of mistreatment, a lot of humiliation. Then my son was born, he also hit me while I was pregnant, he used to kick my stomach a lot, he’d throw me on the bed, it was always beating, beating."*

*D_9: "What I see is that D loves his father, so much, and it’s been so hard for him to understand why he sometimes yells at me, why he has aggressive behaviors. He’s so little, I feel like D is responsible for taking care of me and I feel like that’s not fair. One time his father started yelling at me, raising his voice at me, D stood between us with his tiny hands, ‘Don’t yell at my mommy’."*

#### Alcohol abuse by the father

Excessive and frequent consumption of alcohol is associated with disruptive and violent behaviors in the family environment.

*L.5: "Yes, I lived with L’s father. At first it was because he started drinking about four years into being together, saying he was drinking because we didn’t have babies, now since I had L his alcoholism has increased, and I decided to separate from him. He answers when he wants to. Today, he hasn’t answered. He answered on the 21 st, I told him L was admitted to the hospital and he said ‘Well, what did you do to him?’ and I told him ‘What would I have done? I take care of him’ and well he hasn’t come to support me. If someone had told me that being a mom would be so painful, I would have never had babies…it hurts so much to see my son like this."*

*C_19: "I got together with this person [partner] and there were many fights, a lot of anger… he used to drink [alcohol] and would make me very angry. He never hit me or anything like that, but it did bother me that he would drink and act very heavily while I was pregnant. I mean, it was a terrible stress, awful, so we would argue and fight all the time, well, it was horrible."*

#### Abandonment by the father

Despite the importance of a father figure for the child’s emotional development and the family, a father’s abandonment due to the child’s disease is common.

*J.L.2: "With his father we decided, mostly I decided, to separate, because well he practically never came, very rarely, or he was drunk or always fighting. I said it wasn’t good for the boy being like this and seeing all of that. I’ve been by myself practically for a year, without any financial support from the father, he provides nothing, he doesn’t come to see him either, nothing. Three weeks ago, his father called, just to ask how he was on a video call, and he asked him ‘My son, how are you?’ and my son scolded me and told me ‘Mom, who gave you permission to talk to that man? No, he’s not my father and I don’t want to talk to him’."*

*D.G-6: "No, he doesn’t live with us, he doesn’t help with anything, no, no. He lives here in the city, I don’t know where, but he doesn’t know he has leukemia, we lost touch. No, he had another family. We lost touch many years ago, we never wanted to look for him, he left 12 years ago…".*

### Onset of disease in the child

One of the most relevant themes in this study was the search for a diagnosis and care for the child. The main findings in this regard have been described elsewhere [27].

#### Feminized care

Most of the children received care from their mothers, both at home and at the hospital.

*J_20: "My husband doesn’t understand me. The first time that my daughter was hospitalized for a long time, my husband didn’t go in to see her even once…not once did he go in to see her…he didn’t have to see them suctioning her, he didn’t have to see her getting her blood drawn for oxygenation, getting blood for analyses, he didn’t have to see a lot of things…but he treated us as if…as if we were ignorant, because he said he knew so much ‘Oh! Why are they doing this to the girl? You don’t know’. It was very hard, so hard…he thought we didn’t know how to take care of her."*

*S.Y_12: "[the dad] treated us like… like we were ignorant because he saw that… oh! Why are they doing this to the girl? You don’t know, it was complicated, it was very difficult that stage with him because of the situation where he thought we didn’t know how to take care of her."*

*E_17: "All her life she [the mother-in-law] has told me that it is my fault, that I don’t know how to take care of her, that I am taking money from my husband, that nothing is wrong with my daughter, that I, when her dad was around, she was fine, and now that her dad is gone, she’s not well. And she would tell me that she was sure I brought her here to the hospital. She says, “Who can guarantee that it is true?” I mean, now that she’s with her family, they’re not going to believe me, but they made me feel bad, because they all turned against me."*

#### Mistreatment by health professionals

During a child’s disease, relationships are established with health professionals, especially physicians and close providers. Differences of opinion are frequent and, sometimes, they result in mistreatment. That is, the adverse experiences of caregivers are joined with the burden faced by health professionals working in palliative care.

Repeated exposure to painful and difficult experiences can contribute to fatigue and burnout symptoms among the healthcare team, including physicians, nurses and social workers. Physicians may be more severely affected and start developing avoidant or rigid coping strategies that may lead to negative encounters with caregivers.

Emotions such as anger, guilt and shame may arise. Health professionals may also have issues separating experiences at the hospital from their personal lives outside of it. Hopelessness and detachment arise when faced with continual loss, resulting in refusal to listen to more stories, provision of partial or inaccurate information, or failure to set boundaries between themselves and the families. As a consequence, health professionals exhibit severe mood changes.

Discussing a previous hospital:

*A.E.16: "I can say that that hospital is horrible, their patients die, the children, because of the lack of attention. Nurses, how can you tell those kinds of nurses that they mistreat children? They don’t pay attention to them. My son was hospitalized there for a month and a half and I can tell you that if I hadn’t gotten him out of there, my son would have died […] ‘If you don’t transfer my son somewhere else at some point today, I swear I’m going to sue’…and [the doctor] tells me ‘You have no reason to act like this.’ I told him, “If I’m acting like this it’s because I know you’re not giving him adequate care.”".*

*L_14: "It was a few days … very difficult because they said [At the hospital] … that’s why we were recommended? Who were we? As if I felt superior, but at no time since we arrived until now have, I ever demanded anything. So, they misbehaved, very poorly overall, and it was more serious…".*

Discussing treatment costs at another hospital:

*J.L.2: "[The doctor said] ‘I’ll give you 5 min for you to get the money’…if I had that amount of money, I would have already bought the medication. I didn’t know what to do. I started crying, the doctor told me ‘If you don’t have the money then I’ll discharge him, and you can take him home. Of course, if something happens to him it’ll be your responsibility, it’ll be your fault’."*

Discussing receiving incomprehensible information at the time of diagnosis:

*V-8: "Because when they discharged her, she had a small head, normal, and the doctor told me ‘It could be a cyst, she doesn’t have a corpus callosum’ but I just thought…in my ignorance I thought, well, there’s something missing in her head, but that’s it, I thought, she’s going to be okay. Then two months later, in March, I noticed that her head was growing, so I would tell my husband ‘Hey, I see the girl, her head is growing’ and he would say ‘You’re crazy, she has a big head because you also have a big head, you know how your dad has a big head too.’ But when I came for a check-up in neonatology, they told me the girl had hydrocephalus and she needed surgery urgently."*

### Consequences of disease

#### Painful procedures

Significant discomfort has been identified in a small but consistent group of children with cancer and their caregivers, who are exposed to difficult events and emotions not only as a result of the potential diagnosis of a terminal illness, but due to a series of disruptive actions like invasive procedures—painful biopsies, lumbar punctures, surgeries, radiations, transplants—that can occasionally lead to severe emotional distress.

In the following three cases, caregivers experienced stress as a result of the need for complex and painful procedures:

*C_7: "About a month later her little eyeball had fallen out, it fell out piece by piece and, well, it’s hard to see that. You can see how…it falls out piece by piece…it’s never the same as when she gets surgery, because you don’t notice how they remove it, but I did see how little by little she was losing pieces of her eye. She has a very deep hole that reaches her palate in her little mouth, that’s why she doesn’t speak very well anymore. Doctors from our health center didn’t want to clean her anymore, they didn’t even want to come inside the house. I was desperate until we got to infectiology, they got it under control cleaning with their tools and tweezers and all that, well, they could get it all the way in, the gauze, to clean it out properly."*

*Z-4: "I told my husband: I didn’t like that. They took out a needle, a really big one with liquid inside, they told her ‘Come, let’s go’, laid her down on a bed where the bump was and injected her with anesthesia. They took out the needle and cut. It was shocking because well she was complaining about the pain…they didn’t wait for [the anesthesia] to take effect, they took the sample and sewed her up. I was desperate, scared, I couldn’t stop crying, I couldn’t even speak. My husband arrived and I told him: ‘It’s cancer’."*

*L-11:"Well, the experience…oh! It’s something I wouldn’t wish on anyone, it’s like a nightmare."*

On occasion, anguishing, recurrent, involuntary and intrusive memories are inevitable.

*C-7: "It’s just that if you talk about it, you relive everything that happened and there are times when I think, well, I won’t remember then. But it’s impossible because it’s still there, I get these memories, even if no one asks, but I’m alone and I get those moments. I wouldn’t wish that on anyone."*

#### Unintentional abandonment/feelings of guilt from other family members

It’s common for families at the hospital to have other healthy children or family members, whom they leave behind to take on the responsibility of actively caring for the child patient. Occasionally, siblings can manifest feelings of abandonment or responsibility for their sick sibling, sometimes even leading to self-harm due to guilt.

*C-7: "[Her brother] asks me ‘Is it my fault, because I used to fight with her? Is it because I scolded her or because we fought with each other?’ He wanted to blame himself for what was happening. I think he also wanted to vent, and he slept with me sometimes, but he cried a lot, he cried so much, he looked for us and well it was very sad because we’ve never gone away."*

Discussing a suicide attempt by her elder daughter, a mother said:

*AP_15: "She tried to commit suicide and well she got here. We were also pilgrimaging around the hospital because when I left here, I went home, started washing clothes, when I went up to hang them to dry, I came down and my daughter told me ‘Mom, I feel bad’ ‘What’s wrong?’ ‘I can’t feel my face’ ‘But what did you do? What did you take?’ ‘I took all my pills’ ‘Why did you do that?’ ‘It’s just that you only pay attention to my sister, and it’s just that I also exist’ ‘Yes, my girl, but no, you shouldn’t have done that’ and she started to get unwell. They thought that there was violence at home. My daughter is in psychiatric treatment. They told me they had to investigate. We were like 8 h outside the prosecutor’s office so I could get a sheet of paper and we left at around one, two in the morning. And then I took her home."*

## Discussion

One of the main conclusions of this study—and shared by previous research—is that PPC services should be collaborative and trauma-informed.

It’s been recognized that exposure to stressful situations, such as ACEs, has a devasting effect on the person due to the potential long-term changes in neurodevelopment and gene expression. Additionally, exposure to adversity during childhood is disproportionate in urban environments, among those with less financial resources, and among those belonging to social minority groups underscoring the need for interventions that mitigate the negative impact of ACEs and promote protective factors in order to achieve health equity. Health interventions should therefore account for how experiences of adversity affect individuals and their families throughout their lives and provide integrated, comprehensive support services [28].

The consequences of unaddressed adversity can be severe: people who have lost family members to suicide constitute a group designated as “survivors,” and it has been estimated that every completed suicide can affect an extended network of up to six individuals for decades [29].

The caregivers in this study carry a cumulative burden shaped by intimate partner violence (IPV), ACEs, and the ongoing stress of caring for a seriously ill child, with direct consequences on the quality of care provided in PPC. Unresolved trauma (manifested as depression, anxiety, post-traumatic stress, or emotional dysregulation) compromises the caregiver's capacity to remain present, communicate with the healthcare team, and provide emotional comfort, with reported rates of post-traumatic stress among parents of seriously ill children ranging between 1.6% and 29.3% [30–32]. Active IPV further limits caregivers' ability to attend appointments, adhere to treatment plans, and make autonomous decisions, while significant ACE histories may affect emotional regulation and parenting behaviors in areas such as pain management and end-of-life communication. Beyond the caregiver-child dyad, healthy siblings may also experience unintentional emotional neglect when caregivers concentrate their resources on the sick child; when this dynamic is compounded by a caregiver's trauma history or experience of violence, the risk of broader family destabilization increases, further affecting the environment in which the sick child receives care. These converging adversities make clear that recognizing and addressing caregiver adversity is not peripheral to pediatric palliative care practice, it is intrinsic to it, and reinforce our central recommendation that PPC services adopt a trauma-informed, family-centered framework that supports not only the child, but every vulnerable member of the family unit.

In the context of paediatric palliative care, caregivers face an additional layer of risk, as they experience increased levels of vicarious stress when sharing the emotional, physical, social, and financial difficulties of others in similar situations [33]. Taken together, this evidence highlights that caregivers—particularly those with histories of adversity—carry a cumulative burden that, if left unaddressed, not only threatens their own well-being but also compromises the quality of care provided to seriously ill children.

Vicarious trauma is a process resulting from empathic involvement with trauma survivors; it can affect any individual, but especially health professionals. The British Medical Association [34] highlights a series of common symptoms among physicians and residents who suffer from this condition, including anger, guilt and shame. Additionally, health professionals with vicarious trauma may remain worried about patients even when outside the hospital or over-identify with them.

According to O’Mahony [35], vicarious exposure to trauma is ubiquitous in palliative care. Some authors have highlighted the short- and long-term psychological sequalae of stress among health professionals, which may manifest in competitiveness, feelings of helplessness in ethically problematic situations, and burnout, more than in the death of child patients per se [36].

Family members can experience extreme stress when faced with a child’s illness, procedures, worsening symptoms, and the imminent risk of death. The rates of post-traumatic stress among parents reportedly fluctuate between 1.6% and 29.3% [30]. Cancer has been considered a traumatic experience for both patients and caregivers, leaving them vulnerable to this disorder [31, 32].

Trauma-informed care (TIC) is both a philosophy for approaching patient care and a set of practical skills to reduce the likelihood of retraumatizing patients who have difficult life histories. From this perspective, institutions that practice TIC approach every person as if they already have trauma. To do so, they create a work environment and systemic culture which prioritizes safety, trust, mutual support, collaboration and stabilization, in order to reduce the probability of traumatization. Given that trauma impacts how individuals search for healthcare services, how they interact with health professionals, and how they respond to treatment, hospital staff can and should recognize the impact that trauma can have on throughout the course of the disease [37].

These kinds of interventions have been positively recognized by the American Academy of Pediatrics [38] and utilized by Substance Abuse and Mental Health Services [19]. They are usually structured into five stages:

1. Create awareness about the prevalence of traumatic events among the general population, with an emphasis on the community receiving care. In this case, caregivers of children with critical health problems suffer, according to the results of this study, important and significant trauma, because requiring palliative care entails facing death or the sequelae of severe illness.
2. Stay alert for signs of the impact of trauma on all subjects, not just caregivers and child patients, but on health professionals.
3. Understand the myriads of strategies that caregivers adopt or ways that they react and respond to traumatic events.
4. Nurture is a compromise to approach trauma with ecological and cultural lenses.
5. Prioritize the empowerment, voice and autonomy of caregivers in all aspects of their child’s care and treatment.

A trauma-informed approach aims to avoid traumatization of caregivers and/or patients, but also of the healthcare team. Healthcare institutions can inadvertently or unintentionally create stressful and toxic environments which interfere with patient healing, provider well-being, and overall satisfaction.

The results suggests that diverse governmental and non-governmental entities can contribute to the prevention of ACEs and to the mitigation of their negative effects. In healthcare, for example, primary prevention can be supported through interventions and supervisions during pregnancy and family visits, in order to strengthen parenting skills. Additionally, screening families who belong to communities at risk of ACEs as part of routine health care for children could provide support and reference to specialized services when needed.

In the case of families in this study, it’d be ideal to provide care for healthy siblings who have experienced unintentional abandonment by parents who prioritize caring for a sick child. Sometimes, healthy siblings may even experience feelings of guilt over the illness or death of the child. In the literature this phenomenon is known as survivor’s guilt, commonly occurring among individuals who have been exposed to or witnessed the death or the disease trajectory of a significant person in their lives [39]. As such, they carry a heavy emotional burden and they develop negative self-perceptions, feeling responsible for the illness or death even when they had no power or influence over the situation [39].

Finally, individualized interventions and mental health care for women and other survivors of intimate partner violence or of violence during childhood could be an effective strategy to reduce the burden of disease caused by violence and encourage caregivers to be in healthier relationships.

### Strengths and limitations

This analysis of ACEs and violence among caregivers emerged unintentionally from a study primarily focused on health pilgrimage in the context of PPC. Therefore, one of the limitations of this study is that some key factors and nuances may be missed in our analysis because it was not originally designed to delve into violence; future research should focus on this phenomenon intentionally and in-depth. Relevant themes include the emotional resources and the social support networks built by caregivers, which could be strengthened to ameliorate emotional distress. Even so, we believe this study provides an informative initial look into this topic, enough to justify the creation of a care model that accounts for the experiences of violence lived by caregivers. Several questions arise: What’s the best time to provide support? How long should an intervention last? How can we combine attention to this issue with grief support? How can we combine support for both providers and caregivers?

The greatest strength of this study is thus bringing the issue to light and, importantly, having been able to provide a respectful space for this population to share their experiences and needs.

## Conclusions

The findings of this study reflect the experiences of primary caregivers of seriously ill children receiving palliative care in Mexico, the vast majority of whom are women. This predominance is not incidental, but reflects deeply rooted gender dynamics in which caregiving responsibilities are disproportionately assigned to women, exposing them to a particular convergence of vulnerabilities, including intimate partner violence and ACEs, that directly impacts their capacity to care and their own well-being [40]. Though one man did participate in this study, he did not report experiencing violence or delayed care for his son, which highlights gender as a key factor for quality of care, for the trajectories towards palliative care, and for the violence reported in this process and throughout life (family violence across the lifespan). Overall, the gendered pattern mirrors broader sociocultural realities documented in the literature and underscores the need for gender-sensitive approaches within PPC services. Even so, future research may deliberately seek to include and amplify the experiences of men acting as caregivers, as their perspectives remain largely underrepresented in this field.

While trauma-informed care offers a useful framework, it is insufficient on its own if it fails to account for the specific forms of violence—intimate partner violence, gender-based violence, and adverse childhood experiences—that shape the caregiving context of these women. This is particularly pressing in the Mexican context, where depression, migraines, and other headache disorders are the most prevalent mental health problems among women, representing nearly a 10% increase in disability-adjusted life years due to mental disorders compared to men aged 10 to 64 [41]. These conditions are primarily linked to violence, insecurity, and poverty, and the lack of resources, autonomy, and accessible mental health services prevents many women from leaving violent domestic environments [42], indirectly and unintentionally exposing children to the consequences of that violence and perpetuating a negative intergenerational cycle. Palliative care, by definition, seeks to alleviate suffering and improve quality of life for both patients and their families; yet this commitment remains incomplete when the suffering of caregivers rooted in ongoing or past violence goes unacknowledged. Recognizing these experiences is not merely an ethical imperative but a clinical one: unaddressed violence compromises caregivers’ emotional capacity, disrupts the family dynamic, and ultimately affects the quality of care that seriously ill children receive. Given that mental health services in Mexico are scarce and underutilized, particularly among those with fewer resources [41], the creation of mental health services specifically designed for caregivers within pediatric palliative care is urgent. Pediatric palliative care teams must therefore move beyond a narrow focus on the child’s medical needs and adopt a comprehensive approach that actively identifies and responds to violence against female caregivers as a core component of practice. This includes the prevention of re-traumatization and vicarious trauma among patients, families, and providers, as well as the development of individualized interventions for survivors of violence, which could reduce the burden of disease on caregivers and help disrupt cycles of intergenerational violence. Doing so would not only strengthen the care provided to children with life-limiting conditions but also honour the dignity of the women who sustain that care, often at great personal cost.

## Supplementary Information

Supplementary Material 1.

## Data Availability

The datasets generated and analysed during the current study are not publicly available, as they consist of in-depth interview recordings containing confidential and personally identifiable information from the participants. The original recordings are held under the custody of the corresponding author. De-identified data were shared with the research team for analysis once participant names had been coded. The clinical data and main characteristics of the interviewed caregivers are presented within this article. The full datasets are available from the corresponding author upon reasonable request.

## Abbreviations

PPC: Pediatric palliative care
ACEs: Adverse childhood experiences
