# Supplementary material for: Silent wounds: violence and adverse experiences in parents caring for terminally ill children in palliative care
Source: BMC Palliat Care. 2026 May 11;25:153. doi: 10.1186/s12904-026-02134-9 (PMC13198043; doi:10.1186/s12904-026-02134-9)
Supplement: Supplementary file 1 — Supplementary Material 1. [file 12904_2026_2134_MOESM1_ESM.docx]

**Supplementary file 1**

**GUIDE INTERVIEWS FAMILIES WITH CASES TREATED IN THE PALLIATIVE CARE SERVICE**

Name

Age

Ethnic group

Tongue

Marital status

Family details (children, relatives you live with)

Work

Domicile (permanent)

Address (temporary)

Contact telephone numbers

Building a Family Chart

**TOPICS**

**1. CONCEPTIONS OF SUFFERING AND CARE**

They are all those ideas that allow us to understand and give meaning to this process of life, which is created from a shared social knowledge within a culture, based on lived experiences and transmitted among the members of a human group (reference and expansion from anthropology in general). Conceptions of the disease guide actions aimed at improving or restoring the state of health.

The conceptions about the disease will take into account the following topics, which, when articulated, will enable people to understand what they conceive about the disease and the ways of caring for themselves. By understanding the disease, one can comprehend the actions or strategies that people seek and implement as forms of care.

Questions to explore this topic:

- What is the health problem for which your family member comes to the HIMFG?
- Do you know what your child has?
- Before the doctors told you what it was that you had, did someone give you another diagnosis, explain what you had, or mention another name?
- Do you know if it has other names?
- What does this disease consist of? Do you know why your family member is suffering from this disease?
- Do you know other people (friends, family, or others) who have the same health problem? How do you see them with their disease? Why are they also sick with the same thing?
- Does your family member have a disability
- Does your family member have any special current needs
- Are there any emotional, social, or economic effects?
- Relationship with religion
- According to your beliefs, what is this condition?

CAUSES

- What caused the health problem in your family member?
  - Why?
- How do you know that it was for that reason?
- Has received opinions from others about the causes (family members, conservators in their community, others)
- How is the cause counteracted, if known?
- Can these causative factors be avoided?
- Have some religious beliefs about the disease
- Sources of information about the disease

SYMPTOMS

- What are the symptoms?
- What are the causes of each symptom?
- How does the disease progress due to the symptoms that your family member presents?
- How do you envision your family member's future with these symptoms?
- What do you do daily to counteract these symptoms?
- There are causes of relief and causes of intensification of symptoms

AFTERMATH

- What has changed because of having this health problem in a family member?
- How has your family member's illness affected you in your daily life, in the economy, at work, at home, in the village, in the community, in the relationship with others, in the way you have fun, etc.?
- How has it affected your body, your physical health?
- What are your beliefs about the consequences of illness and healing?

CARE/HEALING. ORGANIZE IN A CHRONOLOGICAL SEQUENCE THE SEARCH FOR ATTENTION...

- Why did you decide to take your family member to the doctor for the first time?
- What are her beliefs about what she should do herself, what the doctor should do, and what the rest of the family should do to get better?
- What are your beliefs about the behavior of others (doctor, extended family, friends, boss, etc)
- On why it does not improve or why it does with certain behaviors or treatments
- What is the role of the traditional doctor?
- Opinion on the role of allopathic physicians in treatment, concerning their beliefs about health

**2. PILGRIMAGE**

Defined as the "never-ending process of creating and negotiating suffering. It not only involves physiological events, but also the way people describe their condition, which differs from how doctors or family members do so, and this extends beyond the accumulation of events caused by the presence of a condition (Pelaez-Ballestas I, 2006). From the way people define their condition (previous topic) and the ways they cope with the disease over time, it is possible to understand how and why patients engage in a process of negotiating suffering throughout their lives with the disease.

- How did you realize that your family member had this problem?
- What do you think about what has happened to you?
- Stages, processes, cycles, and times
- How have you seen that your family member has faced the symptoms throughout the process
- What have been the consequences over time?
- Healers, have you assisted your sick family member?
- Treatments of each, how to cope or deal with side effects, if any.
- What is the effectiveness of each of the treatments, and why do you think they work?
- About crises, have you had crises?, How would you define it?, How have you faced it, how have you faced it together as a family?
- They have had support, social networks (such as trajectory, changes, configuration-reconfiguration)
- What do you think of what has happened in your life as a result of your relative's illness?
- Why has your family member undergone several treatments, if any?

FORMS OF ATTENTION AND PERSONAL EXPERIENCE THROUGHOUT THE PILGRIMAGE

- Since the problems began, what have been the ways to address your family member's health problem (including all healing alternatives)?
- Who recommended that you visit the curators you have met?
- Which ones, at what time, why did they go, and for what?
- What have been the effects of each form of care?
- How were you and your family member treated by each conservator?
- What were the practices (explorations, intervention on the body, interrogation) in each experience with previous healers? Opinion about these procedures performed on your family member
- About medications or treatments taken, and why they have worked or not?
- About the treatment given by the treating physician and their referrals (psychiatry, general practitioner, pain clinic, etc.).
- You have needed exceptional support (from a doctor, psychologist, or psychiatrist). Why?

HOSPITAL CARE EXPERIENCE

- How long have you been going with your family member to the HIMFG?
- Why did you come to the HIMFG, and who recommended it to you?
- Did you know about the existence of the HIMFG, or did you know about it?
- What has the experience been like inside the hospital since your family member's first consultation?
- What has been your experience with doctors, nurses, social workers, and other people within the hospital?
- How has your experience with the care system been?
- How do you typically get to the hospital, by car, taxi, metro, metrobus, or other means?
- Upon arrival at the hospital, there is someone to help you, in case you need it.
- What is your opinion of the hospital's health professionals?
- What is your opinion about the hospital, its facilities, the way it operates, and the way it cares?
- Do you consider that you are treated well or badly? Why?
- Do you consider that your relative is treated well or poorly? Why?
- How have you treated your other family members?
- Do you understand what doctors and nurses tell you? Do you listen to the recommendations? Why?
- On the economic side, you have changed or have had to make adjustments to your economy since the beginning of your relative's illness
- What has been your experience with the treatments that doctors prescribe for your family member?
- What is a consultation day like?
- What is a day of hospitalization like? How do you feel about the care for you and your family member?
- What is the process like for the internment of your family member?
- Regarding food: Do you eat when you are in the hospital accompanying your family member? Where does he eat?
- How is the interaction with patients, doctors, and other hospital staff?

**RELIGION**

- Do you belong to any church or practice any religion?
- Which one?
- Have you leaned more on religion since your family member has the disease?
- How has religion helped your family member's illness?
- Do you ask God for the health of your family member?
- Do you ask others to pray for the health of a family member?
- To whom, or to whom?
- Is there someone who has spiritually accompanied you and your family member since you learned of the disease?
